# CerS1 but Not CerS5 Gene Silencing, Improves Insulin Sensitivity and Glucose Uptake in Skeletal Muscle

**DOI:** 10.3390/cells11020206

**Published:** 2022-01-08

**Authors:** Agnieszka U. Błachnio-Zabielska, Kamila Roszczyc-Owsiejczuk, Monika Imierska, Karolina Pogodzińska, Paweł Rogalski, Jarosław Daniluk, Piotr Zabielski

**Affiliations:** 1 Department of Hygiene, Epidemiology and Metabolic Disorders, Medical University of Bialystok, Mickiewicza 2c, 15-089 Bialystok, Poland; kamila.roszczyc-owsiejczuk@umb.edu.pl (K.R.-O.); m.imierska@gmail.com (M.I.); karolina.pogodzinska@umb.edu.pl (K.P.); 2 Department of Gastroenterology and Internal Medicine, Medical University of Bialystok, 15-089 Bialystok, Poland; progalsky@gmail.com (P.R.); jaroslaw.daniluk@umb.edu.pl (J.D.); 3 Department of Medical Biology, Medical University of Bialystok, 15-089 Bialystok, Poland

**Keywords:** skeletal muscle, insulin resistance, ceramides, gene silencing, mass spectrometry

## Abstract

Skeletal muscle is perceived as a major tissue in glucose and lipid metabolism. High fat diet (HFD) lead to the accumulation of intramuscular lipids, including: long chain acyl-CoA, diacylglycerols, and ceramides. Ceramides are considered to be one of the most important lipid groups in the generation of skeletal muscle insulin resistance. So far, it has not been clearly established whether all ceramides adversely affect the functioning of the insulin pathway, or whether there are certain ceramide species that play a pivotal role in the induction of insulin resistance. Therefore, we designed a study in which the expression of CerS1 and CerS5 genes responsible for the synthesis of C18:0-Cer and C16:0-Cer, respectively, was locally silenced in the gastrocnemius muscle of HFD-fed mice through in vivo electroporation-mediated shRNA plasmids. Our study indicates that HFD feeding induced both, the systemic and skeletal muscle insulin resistance, which was accompanied by an increase in the intramuscular lipid levels, decreased activation of the insulin pathway and, consequently, a decrease in the skeletal muscle glucose uptake. CerS1 silencing leads to a reduction in C18:0-Cer content, with a subsequent increase in the activity of the insulin pathway, and an improvement in skeletal muscle glucose uptake. Such effects were not visible in case of CerS5 silencing, which indicates that the accumulation of C18:0-Cer plays a decisive role in the induction of skeletal muscle insulin resistance.

## 1. Introduction

Obesity is associated with a number of metabolic disorders, including cardiovascular disease, atherosclerosis, insulin resistance, and type 2 diabetes (T2D) [1,2]. This condition is accompanied by an increased concentration of plasma free fatty acids (FFA) [3]. Skeletal muscle is an important tissue involved in the regulation of both, glucose and lipid metabolism. The availability of FFAs is very important in lipid metabolism as they are substrates for de novo lipid synthesis. Plasma FFAs enter muscle by passive diffusion or via protein transporters: the fatty acid translocase (FAT/CD36) [4], fatty acid binding protein (FABPpm) [5], and fatty acid transport protein 1 (FATP1). FAs that enter the cell are converted to a long-chain acyl-CoA (LCACoA in a reaction catalyzed by acyl-CoA synthetase [6]. LCACoA can be used as substrates in the de novo synthesis of other lipids or can be directed to the mitochondria for β-oxidation [7]. In the transport of LCACoA across the mitochondrial membrane, an important role is played by carnitine palmitoyltransferase 1 (CPT1), which catalyzes the transfer of acyl group from LCACoA to L-carnitine, resulting in the formation of acylcarnitines. The increased FFA uptake and decreased β-oxidation rate contributes to the accumulation of intramyocellular lipids. Among these lipids are also biologically active ones such as ceramides (Cer), diacylglycerols (DAG), and LCACoA [8,9,10,11,12]. Each of these lipid groups negatively affects the insulin signaling at different levels of its intracellular signaling pathway. However, for several years, researchers’ attention regarding muscle insulin resistance has focused on ceramides. The increased intracellular ceramide content was observed in the muscles of HFD-fed animals [13,14], obese, insulin resistant Zucker rats [15], and obese insulin resistant people [16]. The increased ceramide level was accompanied by a decrease in muscle insulin sensitivity. It has been repeatedly shown that ceramide inhibits the insulin pathway at the level of protein kinase B Akt/PKB by activating phosphatase A2 (PPA2), which keeps PKB in an unphosphorylated state [17]. However, it has not yet been clearly established whether there is one specific species or a related group of ceramides that plays a critical role in inducing dysfunction in the insulin pathway. De novo ceramides synthesis is a multi-stage process. The first step of this process begins with the transfer of palmitate from palmityl-CoA to serine, catalyzed by serine palmityl transferase. After reduction to sphinganine, the next step in which another acyl-CoA is attached is a reaction catalyzed by ceramide synthase (CerS). CerSs are responsible for the acylation of sphingoid base, sphingosine, or sphinganine, with fatty acids of various chain lengths, yielding ceramide or dihydroceramide, respectively. So far, six isoforms of this enzyme have been discovered and each CerS has a distinct substrate specificity towards the acyl-CoA chain length. In skeletal muscle, the highest expression was found for CerS1 and CerS5, which are responsible for the generation of C18:0-Cer and C16:0-Cer, respectively [18]. In our previous work, we showed that HFD leads to the highest increase in C18:0-Cer, which was accompanied by a weakening of the insulin signaling pathway, and the administration of myriocin causes a decrease in ceramide levels, with the largest decrease in C18:0-Cer, accompanied by an improvement in the function of the insulin signaling pathway in muscles [13]. This discovery was also confirmed by other researchers who showed that the deletion of CerS1 and, hence, decrease in C18:0-Cer levels in skeletal muscles, improves systemic glucose metabolism in obesity [19]. However, the results do not overlap with another group of researchers who have shown that the use of a CerS1-specific inhibitor increases the rate of fatty acid oxidation in muscle, but does not protect against diet-induced insulin resistance [20]. Moreover, some researchers postulate that the accumulation of C16:0-Cer is responsible for inducing insulin resistance. Therefore, we designed our research to understand which of these ceramides play a dominant role in disrupting the insulin pathway. To achieve this goal, we investigated the effect of local in vivo shRNA-mediated gene silencing of CerS1 and CerS5 on the bioactive lipid accumulation and insulin signaling pathway in gastrocnemius muscle of mice with diet-induced insulin resistance.

## 2. Materials and Methods

The experiment was approved by the Local Ethical Committee for Animal Experiments (Olsztyn, Poland, approval number 43/2016). The studies were performed on six-week old male C57BL/6 mice (20 g) (Jackson Laboratory; Bar Harbor, ME, USA). The animals were housed in 12 h light/dark cycle with free access to food and water. Mice were randomly divided into following groups: Control group (*n* = 8), fed a control low fat diet (LFD), with the both hindlimb gastrocnemius muscles injected with scrambled shRNA plasmid, HFD animals (*n* = 8), fed HFD with one hindlimb gastrocnemius muscle electroporated with shRNA plasmids targeted towards CerS1 (HFD_(-CerS1)_) and the opposed hindlimb gastrocnemius muscle electroporated with scrambled shRNA plasmid (HFD_(+/+)_) and HFD animals (*n* = 8), fed HFD with one hindlimb gastrocnemius muscle electroporated with shRNA plasmids targeted towards CerS5 (HFD_(-CerS5)_) and the opposed hindlimb gastrocnemius muscle electroporated with scrambled shRNA plasmid (HFD_(+/+)_). All animals were fed for 8 weeks with the appropriate diet. The LFD was composed of 70% carbohydrate, 10% fat, and 20% protein (% energy) (Research Diets INC D12450J) while HFD was composed of 20% carbohydrate, 60% fat, and 20% protein (% energy) (Research Diets INC, D12492). Two weeks before sacrifice, an oral glucose tolerance test (OGTT) was performed, and one week later, an insulin tolerance test (IPTT) was performed. Exactly 20 min prior to euthanasia, 2-deoxy-[1.2-3H (N)]-D-glucose (12 µCi/25 g of fat-free mass, in 25 µL of PBS, Perkin-Elmer, MA) was administered via the lateral tail vein followed by intraperitoneal injection of insulin at a dose of 0.5 U/kg to measure insulin-stimulated muscle glucose uptake and protein phosphorylation of the insulin pathway The mice were euthanized by cervical dislocation, gastrocnemius muscles were taken, frozen in liquid nitrogen, and then stored at −80 °C until analysis.

### 2.1. Plasmids and In Vivo Electroporation

Bacterial stocks of plasmids producing the appropriate shRNA/TurboGFP scrambled shRNA sequences were purchased from Dharmacon (currently Horizon Discovery, Cambridge, UK). After amplifying the plasmids in bacterial culture according to the manufacturer’s guidelines, the plasmids were isolated and purified using the GeneJET Plasmid Maxiprep Kit (Thermo Scientific), and stored in −80 °C. A mixture of plasmids encoding 3 different shRNA sequences against the target gene was prepared in 150 mM PBS (pH = 7.2). After two weeks of acclimatization and feeding with the appropriate diet, mice were anesthetized in an induction chamber with ~2% isoflurane in oxygen using a UNO BV rodent anesthesia system (UNO, Zevenaar, Holland).

**Figure 1:**
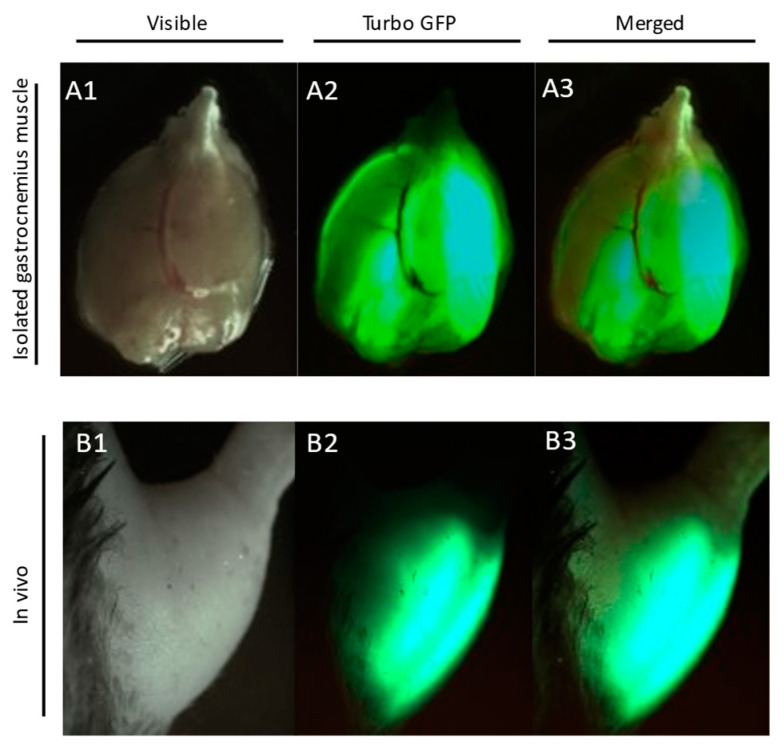
TurboGFP reporter gene expression in gastrocnemius muscle. Images were taken at 6 weeks after plasmid transfection: Panel **A1**—visible light photo of isolated mouse gastrocnemius muscle; Panel **A2**—Turbo-GFP fluorescence of electroporated gastrocnemius; Panel **A3**—**A1** and **A2** merged; Panel **B1**—visible light photo of mouse hindlimb; Panel **B2**—transcutaneous Turbo-GFP fluorescence of electroporated gastrocnemius; Panel **B3**—**B1** and **B2** merged.

During the electroporation procedure, mice were kept on a heating blanket under general isoflurane anesthesia. Prior to plasmid administration, the area above the gastrocnemius muscle was shaved and 30 µL of hyaluronidase (0.4 U/µL) was injected in two divided doses of 15 µL on both sides of the muscle. Two hours later, 40 µL (2 µg/1 µL) of plasmid solution was injected into the gastrocnemius muscle. Electrical pulses (175 V/cm, 8 pulses, 200 ms intervals) were applied using a pair of plate electrodes (area 1 cm^2^) placed on each side of the muscle [21]. A plasmid containing the scrambled shRNA sequences was injected into the muscle of the other leg. GFP reporter gene expression in both legs was monitored transcutaneously once a week using a UV flashlight (Figure 1).

### 2.2. Lipid Measurements

#### 2.2.1. Plasma FFA

The concentration of plasma FFA was measured with the use of LC/MS according to Persson et al. [22]. Briefly, a known amount of C14:0-d27, C15:0, C16:0-d31, C17:0, C18:1-d9, and C18:0-d35 was added to each plasma sample as an internal standard. The samples were extracted with Dole solution composed of isopropanol: heptane:1 M H_2_SO_4_ (40:10:1; *v*/*v*/*v*). The extracted samples were then evaporated under a stream of nitrogen and suspended in buffer A for LC/MS analysis (Sciex QTRAP 6500+, AB Sciex Germany GmbH, Darmstadt, Germany). Chromatographic separation of fatty acids was performed with the use reverse phase column (2.1 × 150 mm, 1.8 µm Zorbax SB-C18) using two buffers: buffer A—80% acetonitrile, 0.5 mM ammonium acetate; buffer B was 99% acetonitrile, 1% 0.5 mM ammonium acetate. FFA concentrations were measured against a six-point standard curve prepared for each compound.

#### 2.2.2. Sphingolipids

The sphingolipids content were measured with the use of UHPLC/MS/MS according to Blachnio-Zabielska et al. [23] with minor modifications. Briefly, muscle samples (~20 mg) were pulverized and then homogenized in a buffer consisting of 0.25 M sucrose, 25 mM KCl, 50 mM Tris, and 0.5 mM EDTA, pH 7.4. Immediately afterwards, a mixture of internal standards (Sph-d7, SPA-d7, S1P-d7, C15:0-d7-Cer, C16:0-d7-Cer, C18:1-d7-Cer, C18:0-d7-Cer, 17C/20:0-Cer, C24:1-d7-Cer, C24-d7-Cer Avanti Polar Lipids, Alabaster, Al) and extraction mixture (isopropanol:water:ethyl acetate, 30:10:60); *v*:*v*:*v*) have been added to each sample. After extraction, the samples were evaporated under a stream of nitrogen and suspended in LC Solvent B (2 mM ammonium formate, 0.1% formic acid in methanol) for UHPLC/MS/MS analysis. The chromatographic separation was performed using a reverse-phase Zorbax SB-C8 column 2.1 × 150 mm, 1.8 μm (Agilent Technologies, Santa Clara, CA, USA) in binary gradient using 1 mM ammonium formate, 0.1% formic acid in water as solvent A, and 2 mM ammonium formate, 0.1% formic acid in methanol as solvent B at the flow rate of 0.4 mL/min. Sphingolipids were analyzed using Sciex QTRAP 6500 + triple quadrupole mass spectrometer (AB Sciex Germany GmbH, Darmstadt, Germany) with multiple reaction monitoring (MRM) against standard curves constructed for each compound.

#### 2.2.3. Diacylglycerols

The DAG content in skeletal muscle was analyzed with the use of UHPLC/MS/MS according to Blachnio-Zabielska et al. [24]. The compounds were extracted together with sphingolipids. Prior to extraction, a known amount of an internal standard mixture (Deuterated DAG Mixture I and Mixture II—Avanti Polar Lipids) was added to each sample. The content of diacylglycerols (C16:0/18:2, C16:0/16:0, C16:0/18:1, C18:0/20:0, C18:0/18:1, C18:1/18:1, C18:2/18:2, C18:0/18:0, C18:0/18:2, and C16:0/18:0) was determined using a triple quadrupole mass spectrometer (Sciex QTRAP 6500 + AB Sciex Germany GmbH, Darmstadt, Germany). against standard curves prepared for each compound.

#### 2.2.4. Long-Chain Acyl-CoA

The content of LCA-CoA was measured according to Blachnio-Zabielska et al. [25]. Briefly, acyl-CoA was extracted according to Minkler et al. [26]. Before extraction, a known amount of the internal standards mixture (C15:0-CoA, 16:0(d4)-CoA, C17-CoA, C19:0-CoA, C21:0-CoA, C23:0:-CoA, and 24:0(d4) CoA) was added to each sample. Acylo-CoAs (C14:0-CoA, C16:0-CoA, C16:1-CoA, C18:2-CoA, C18:1-CoA, C18:0-CoA, C20:0-CoA, C22:0-CoA, C24:1-CoA, C24:O-CoA) were separated on an Agilent ZORBAX Extend-C18 reverse phase column 2.1 × 150 mm, using a binary gradient with ammonium hydroxide (NH_4_OH) in water and NH_4_OH in ACN. The acyl-CoA content was determined with a triple quadrupole mass spectrometer (Sciex QTRAP 6500 + (AB Sciex Germany GmbH, Darmstadt, Germany) against standard curves prepared for each compound.

#### 2.2.5. Acyl-Carnitines

Acyl-carnitine content was determined according to Giesbertz [27] with minor modification. Briefly, muscle samples were pulverized, then an internal standard (C17-carnitine) was added to each sample, extracted with methanol, and centrifuged at 10,000 g, 4 °C for 10 min. The supernatants were evaporated under a stream of nitrogen. The acylcarnitines were then derivatized to their butyl esters by incubation with n-butanol containing 5% *v*/*v* acetyl chloride. The samples were then evaporated under a nitrogen stream and suspended in 100 µL of a methanol/water. Chromatographic separation was performed on a 2.1 × 150 mm, 1.8 µm reverse phase Zorbax SB-C18 column (Agilent Technologies, Santa Clara, CA, USA) by ultra-high performance liquid chromatography (Shimadzu Nexera-X2 UHPLC). Quantitative analyzes of acyl-carnitines were determined with the use of Sciex QTRAP 6500 + triple quadrupole mass spectrometer (AB Sciex Germany GmbH, Darmstadt, Germany) using electrospray ionization (ESI) with multi-reaction monitoring (MRM) against standard curves prepared for each compound.

#### 2.2.6. Triacylglycerols

Muscle triacylglycerol content was analyzed with the High Sensitivity Triglyceride Fluorometric Assay Kit (Sigma Aldrich, MAK264-1KT), according to manufacturer’s protocol.

### 2.3. Western Blotting

Muscle samples were homogenized in RIPA buffer (Sigma-Aldrich) with 0.5 mM TCEP (Sigma-Aldrich) and protease and phosphatase inhibitors. Content of protein in homogenates was measured using Pierce 660 nm protein assay kit (Thermo Fisher Scientific, Waltham, MA, USA). Fatty acid free bovine serum albumin was used as a standard. After denaturation in Laemmli buffer, proteins were separated by SDS-PAGE (BioRad Criterion Cell) and transferred to a PVDF membrane (BioRad Trans Blot SD semi-dry transfer cell with a discontinuous Tris/CAPS buffer system, 15% methanol for the anode and Tris/CAPS 0.1% SDS for the cathode). After blocking with Every Blot Blocking Buffer (Bio Rad), membranes were incubated with the appropriate primary antibody. Following target proteins were quantified using rabbit primary antibodies: CD36 (NB400-144, Novus Biologicals), FATP1 (H00376497-D01P, Novus Biologicals), glucose transporter 4 (GLUT4) (ab33780), IR (ab5500), pIR(Y972) (ab5678), FABPpm, (ab153924), creatinine-O-palmitoyltransferase 1 (CPT1) (ab104662) (Abcam, Cambridge, MA), Akt (4691S), pAktSer473 (4060P), IRS1 (2390), pIRS1(Ser1101) (2385), AS160 (2670S), AS160Ser588 (8730S) (Cell Signaling Technology), and glyceraldehyde 3-phosphate dehydrogenase (GAPDH) (ab9485) (Abcam, Cambridge, MA). To detect primary antibody binding to protein, the membrane was incubated with an HRP-conjugated secondary antibody (ab6885) followed by a Clarity™ Western ECL chemiluminescent substrate (Bio-Rad) and visualized using a Bio-Rad ChemiDoc XRS+ imaging system. Band intensities were quantified with the Bio-Rad Image Lab software package. The obtained values were normalized to the GAPDH protein measured from parallel runs and expressed as fold changes over control group value. Unless otherwise stated, all chemicals and equipment used for immunoblotting were purchased from Bio-Rad (Hercules, CA, USA).

### 2.4. Real-Time PCR

Total RNA was isolated from muscle samples using the mirVana Isolation Kit (Thermo Fisher Scientific, USA) according manufacturer instruction. The isolated RNA was then reverse transcribed into cDNA using Transcriptor First Strand cDNA Synthesis Kit (Roche). Real-time PCR was performed with RealTime ready SYBR Green Assays (Roche Mannheim, Germany) prepared for CerS1, CerS5, and GAPDH, which was used as a housekeeping gene using a LightCycler480 system (Roche Mannheim, Germany). The results were normalized to GAPDH expression measured in each sample.

### 2.5. Insulin-Stimulated Glucose Uptake

Skeletal muscle glucose uptake under insulin stimulation was measured with the intravenous bolus of 2-deoxy-[1,2-3H (N)]-D-glucose, as described earlier [28]. Animals received radiolabeled glucose injection through lateral tail vein 20 min prior to euthanasia (12 µCi/25 g fat-free mass, total volume approx. 25 µL, Perkin-Elmer, Waltham, MA, USA). Subsequently, it was followed by intraperitoneal injection of insulin (0.5 U/kg of fat-free mass). Blood (25 µL) was collected at 2.5, 5, 7.5, 10, 12.5, and 15 min after radiotracer/insulin injection to estimate the 2-deoxy-[1,2-3H (N)]-D-glucose enrichment. Specific activity 2-deoxy-[1,2-3H (N)]-D-glucose in plasma and skeletal muscle was measured with the use of 0.3 N Ba(OH)_2_/0.3 N ZnSO_4_ precipitation and liquid scintillation counter (LSC). Skeletal muscle samples were sonicated in 6% HClO_4_, neutralized with 5 M KOH and fractionated into 2-deoxy-D-glucose and 2-deoxy-D-glucose-6-phosphate with 0.3 N Ba(OH)_2_/0.3 N ZnSO_4_. Plasma samples were deproteinized with 0.3 N Ba(OH)_2_/0.3 N ZnSO_4_. Radioactivity was estimated with Packard Tri-Carb 1900TR LSC. Tissue-bound 2-deoxyglucose-6-phosphate was calculated as difference in radioactivity in the HClO_4_ and Ba(OH)_2_/ZnSO_4_ tissue extracts. Insulin stimulated glucose uptake was calculated using the equation devised by Fueger et al.: Rg = [Cm∗(T)/$\int_{0}^{T}\left(Cp*/Cp\right)$] *∆*t* [29], where *Cm*∗(*T*)—Tissue radioactivity of 2-deoxy-[1,2-3H (N)]-D-glucose/mg at the end of experiment (DPM/mg), ∆*t*—Time from glucose bolus injection to tissue collection (min), $\int_{0}^{T}\left(Cp*/Cp\right)$—Area under plasma glucose enrichment curve calculated with the use of trapezoidal rule (DPM/mg/min).

### 2.6. Plasma Insulin and Glucose Concentration

Both plasma insulin and blood glucose were measured in mice that were fasted for six hours. Insulin concentration was measured using an ELISA insulin assay (Rat/Mouse Insulin Millipore). Plasma glucose concentration was determined with the use of an AccuChek Aviva glucometer (Roche, Germany).

### 2.7. Oral Glucose Tolerance Test (OGTT)

On the day of experimentation, mice were fasted for six hours and an OGTT was performed using a 2 g/kg. Blood glucose was measured at 15, 30, 60, 90, and 120 min after oral glucose administration. Area under glucose concentration curve was measured with the use of trapezoidal rule.

### 2.8. Insulin Tolerance Test (ITT)

Mice were fasted for six hours and were injected intraperitoneally with insulin at a dose of 0.75 U/kg. Glucose levels were measured in blood samples using an AccuChek Aviva glucometer (Roche, Germany) at 0, 15, 30, 45, 60, 90, and 120 min after the insulin injection. Area under glucose concentration curve was measured with the use of trapezoidal rule.

### 2.9. HOMA-IR (Homeostatic Model Assessment)

HOMA-IR index value was calculated according to formula [30].

HOMA-IR = (fasting glucose (mg/dl) × fasting insulin (μIU/mL))/2430.

### 2.10. Statistical Analysis

Results were expressed as mean +/− standard deviation (*n* = 8 per group, if not stated otherwise). For multiple group testing, significant differences were identified by one-way ANOVA with Tukey Honest Significant Difference post-hoc test (Tukey HSD) for equal group size. For two group testing, significance was identified by unpaired t-test. Significance threshold was set at *p* < 0.05 in both cases.

## 3. Results

### 3.1. Gene Silencing of CerS1 and CerS5

**Figure 2:**
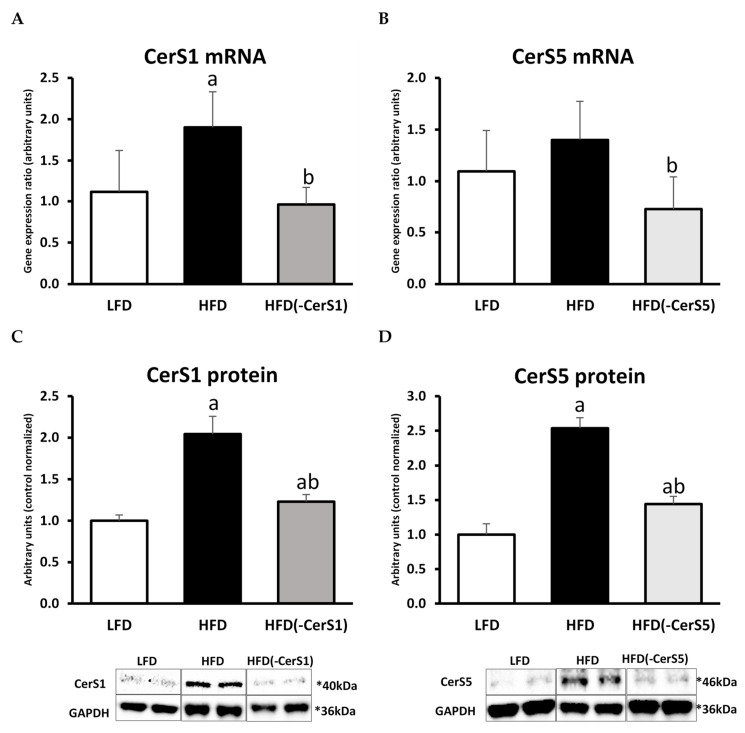
The impact of in vivo CerS1 and CerS5 gene silencing on the mRNA and protein expression of CerS1 and CerS5 in the mouse gastrocnemius muscle: Panels (**A**,**C**)—the expression skeletal muscle CerS1 at the mRNA and protein level, respectively; Panels (**B**,**D**)—the expression skeletal muscle CerS5 at the mRNA and protein level, respectively. Values are mean ± SD (*n* = 8 per group); a—*p* < 0.05 vs. LFD; b—*p* < 0.05 vs. HFD; significance by ANOVA; LFD-low-fat diet, HFD—high-fat diet. The observed molecular weight of indicated proteins is different to the theoretical one, as stated by the antibody manufacturer.

The CerS1 mRNA level significantly increased in the gastrocnemius muscle of HFD-fed animals (by 71%, *p* < 0.05) as compared to control LFD animals and decreased (by 50%, *p* < 0.05) in the HFD_(-CerS1)_ muscle as compared to HFD_(+/+)_. Similar pattern was observed in the protein content of CerS1 (Figure 2A,C).

The content of CerS5 mRNA increased in HFD_(+/+)_ muscle (by about 28%, *p* < 0.05) compared to LFD group. In the muscle of HFD_(-CerS5)_ the mRNA decreased by about 48% (*p* < 0.05) as compared to HFD_(+/+)_. The level of CerS5 protein in the HFD group was 2.5 times higher than in the control LFD group. CerS1 gene silencing in the muscle of animals fed HFD caused a decrease in the content of its protein product by about 42% compared to HFD_(+/+)_ animals (Figure 2B,D).

HFD consumption resulted in a significantly higher up-regulation of CerS1 mRNA and protein compared to the same values observed for CerS5 isoform. Silencing of CerS genes in skeletal muscle via electroporation-mediated shRNA plasmid transfection was equally effective for both the isoforms. The observed changes in the content of CerS1 and CerS5 caused by HFD and/or silencing of individual genes were reflected in the ceramide content in these muscles. In the HFD_(+/+)_ group, we noticed an increase in the level of all measured ceramides, with the highest elevation observed for C18:0-Cer and C24:0-Cer species. The most abundant ceramide species in skeletal muscles is C18:0-Cer, and its content largely determines the total muscle ceramide level. In the muscle of HFD_(-CerS1)_ animals, we observed about 40–45% decrease in the content of C18:1-Cer and C18:0-Cer. Accordingly, CerS5 inhibition resulted in significant HFD_(-CerS5)_ decrease in C16:0-Cer, but because this ceramide species displays significantly smaller fraction of total muscle Cer than 18-carbon species counterparts, the overall drop in total ceramide concentration in HFD_(-CerS5)_ muscle was less pronounced than in HFD_(-CerS1)_ counterpart.

### 3.2. The Effect of HFD on Glucose and Insulin Concentration and HOMA-IR Value

**Figure 3:**
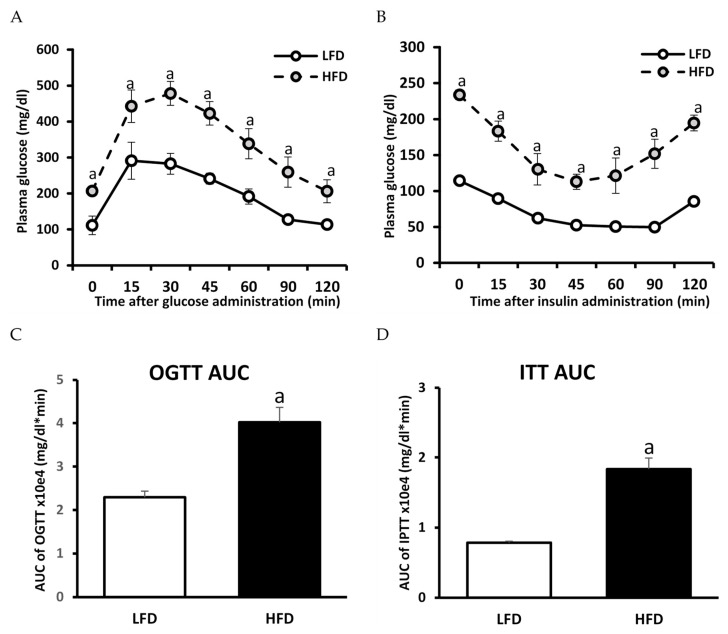
Impact of high-fat diet (HFD) consumption on plasma glucose profile during oral glucose tolerance test (OGTT) and intraperitoneal insulin tolerance test (ITT): Panel (**A**)—plasma glucose profile during OGTT; Panel (**B**)—plasma glucose profile during IPTT; Panel (**C**)—area under the plasma glucose curve (AUC) for OGTT; Panel (**D**)—area under the plasma glucose curve (AUC) for IPTT. Values are mean ± SD (*n* = 6 per group); a—*p* < 0.05 vs. LFD; significance by *t*-test.

Mice fed HFD displayed an increase in the concentration of plasma fasting glucose and insulin. As a result of these changes, an increase in the HOMA-IR index was observed in HFD-fed animals as compared to the LFD ones. Moreover, OGTT and ITT showed an impaired glucose tolerance as well as diminished insulin response in animals fed HFD diet as compared to LFD animals, demonstrating the induction of insulin resistance in HFD animals (Table 1; Figure 3). Figure S1 shows baseline-normalized plasma glucose curves.

### 3.3. Plasma FFA Concentration

Total plasma FFA concentration increased significantly in the HFD animals (by 89%; *p* < 0.05) as compared to LFD. Almost all fatty acids increased in the HFD group (except palmitoleic acid C16:1 and eicosadienoic acid C20:2) as compared to LFD control. The highest increase was observed for arachidonic C20:0 and behenic C22:0 fatty acids (*p* < 0.05, Table 1 and Table S1).

### 3.4. Skeletal Muscle Fatty Acid Transporters

#### 3.4.1. CD36

The protein content of CD36 significantly increased in the HFD_(+/+)_ muscle as compared to LFD control. In the gastrocnemius muscle of both, HFD_(-CerS1)_ and HFD_(-CerS5)_ animals, a decrease in CD36 protein content was observed as compared LFD control (Figure S2A).

#### 3.4.2. FATP1

In the HFD_(+/+)_ gastrocnemius muscle, the level of FATP1 protein significantly increased as compared to LFD control. The CerSs genes silencing (CerS1 and CerS5) significantly decreased FATP1 protein content as compared to HFD_(+/+)_, but its content was still higher than LFD control values (Figure S2B).

#### 3.4.3. FABPpm

The changes in FABPpm protein content were similar to those observed for CD36 and FATP1 transporters. In the muscle of HFD_(+/+)_ animals, the FABPpm protein content significantly increased as compared to LFD control and decreased in both, HFD_(-CerS1)_ and HFD_(-CerS1)_ silenced muscles as compared to HFD_(+/+)_ (Figure S2C).

### 3.5. Lipids

#### 3.5.1. LCACoA

**Figure 4:**
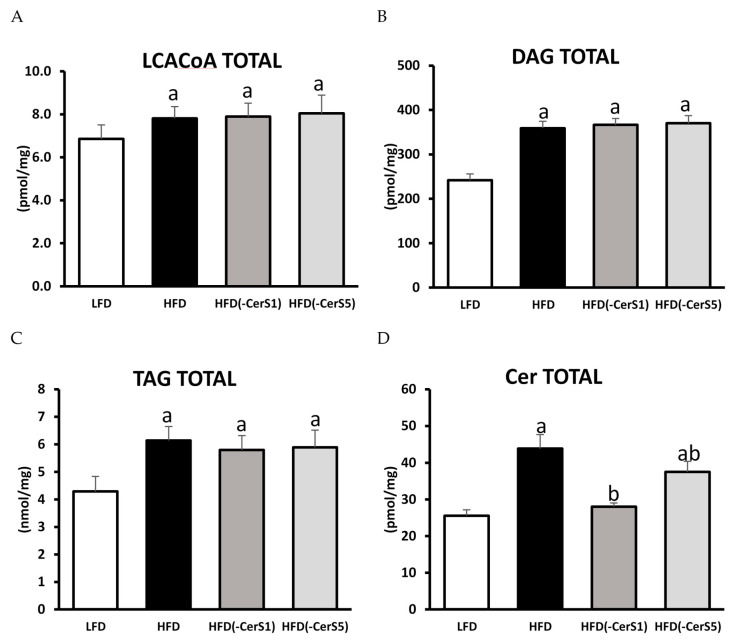

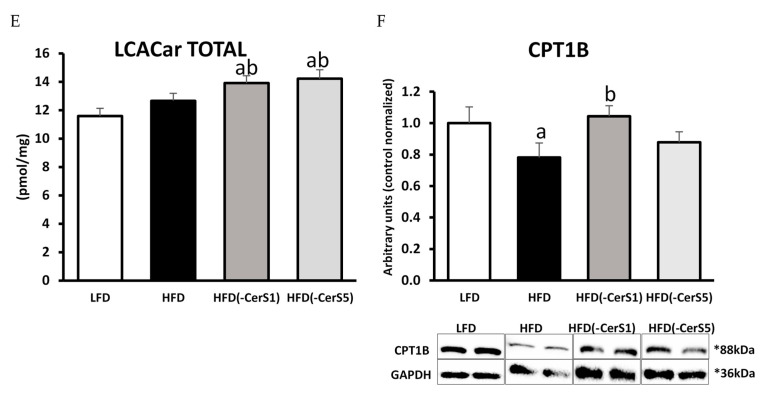
The impact of CerS1 and CerS5 silencing on the content of bioactive lipids, acyl-carnitines, and the protein expression of CPT1B in gastrocnemius muscle of HFD-fed mice: Panel (**A**)—total content of LCACoA; Panel (**B**)—total content of DAG; Panel (**C**)—total content of TAG; Panel (**D**)—total Cer content; Panel (**E**)—total content of acyl-carnitines, Panel (**F**)—protein of CPT1B. Values are mean ± SD (*n* = 8 per group); a—*p* < 0.05 vs. LFD; b—*p* < 0.05 vs. HFD; significance by ANOVA; LFD-low-fat diet, HFD—high-fat diet. The observed molecular weight of indicated proteins is different to the theoretical one, as stated by the antibody manufacturer.

Regardless of gene silencing, in the muscles of all HFD-fed groups, a similar, statistically significant increase in total LCACoA content was observed. Major molecular species responsible for the observed increase were C14:0-CoA, C18:0-CoA, C22:0-CoA, and C24:1-CoA (Figure 4A; Table 2).

#### 3.5.2. DAG and TAG

The total content of both DAG and TAG increased significantly in the muscles of HFD_(+/+)_ animals, irrespective of gene silencing, as compared to LFD control. Among DAGs, the highest increase was recorded for 16:0/18:0-, 16:0/18:2-, 18:0/18:2-, 18:2/18:2-, and 18:0/20:0-DAG (Figure 4B,C; Table 3).

#### 3.5.3. Sphingolipids

Sphinganine (SPA), sphingosine (Sph), and sphingosine-1-phosphate (S1P) as well as total ceramide content significantly increased in HFD_(+/+)_ gastrocnemius muscle as compared to LFD control (*p* < 0.05). HFD feeding resulted in the increase in the content of almost all measured ceramide species, except for C14:0-Cer. Silencing of the CerS1 gene in the gastrocnemius muscle of HFD-fed mice resulted in a significant decrease in the level of S1P and total ceramide as compared to the HFD_(+/+)_ animals (*p* <0.05). Significant drop in the content of C18:0-Cer, which is the most abundant ceramide species in skeletal muscle, was responsible for the overall decrease in the muscular Cer in the HFD_(-CerS1)_ animals. In the gastrocnemius muscle of HFD_(-CerS5)_ animals, there was a significant decrease in the total level of ceramide compared to the HFD_(+/+)_ animals, yet it was still higher than in the LFD control group. In this group, the highest decrease was observed in the C16:0-Cer species (Figure 4D, Table 4).

#### 3.5.4. Acyl-Carnitines

Although HFD feeding had no significant effect on the muscular acyl-carnitine content, both the CerS1 and CerS5 silencing lead to significant elevation in the quantity of acylated carnitine species, as compared to both the LFD control and HFD-only animals (Figure 4E, Table S2).

### 3.6. CPT1

CPT1 protein content significantly decreased in the HFD_(+/+)_ gastrocnemius muscle as compared to the LFD control (*p* < 0.05). Interestingly, the protein content of CPT1 in HFD_(-CerS1)_ gastrocnemius increased significantly as compared to HFD_(+/+)_ muscle, whereas CerS5 silencing yield no significant changes at the level of CPT1 protein level, neither compared to the LFD nor HFD_(+/+)_ animals (Figure 4F).

### 3.7. Insulin Signaling Pathway

#### 3.7.1. Insulin Receptor (IR)

**Figure 5:**
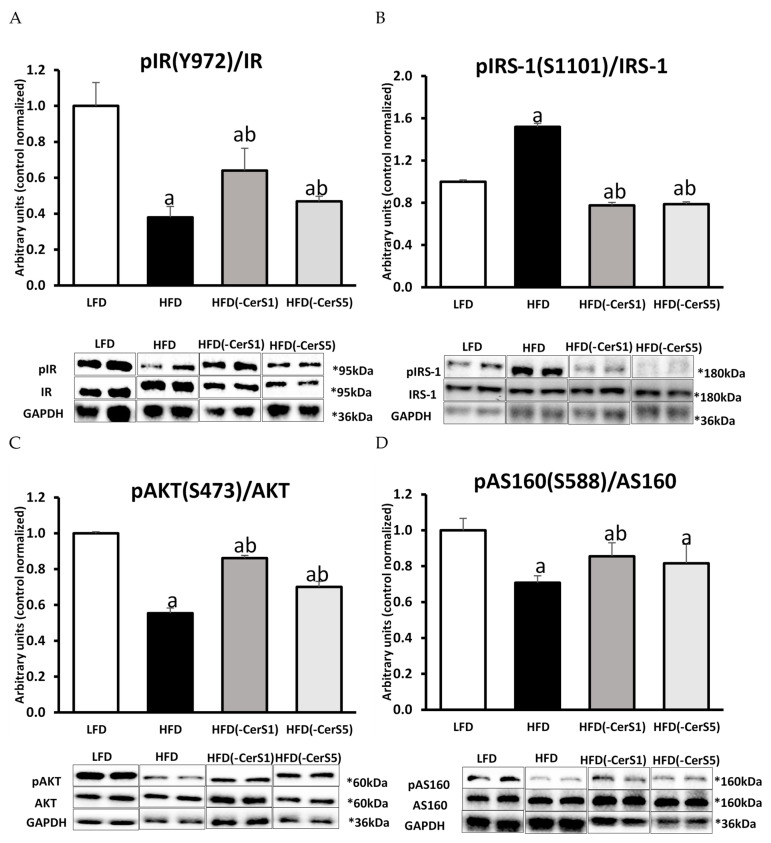

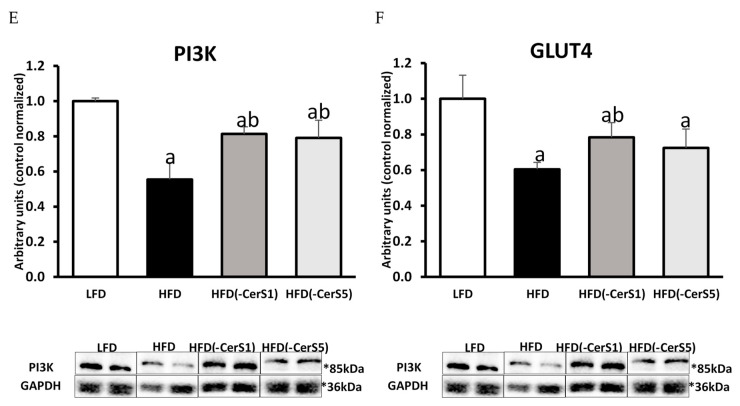
Activation of insulin signaling pathway in CerS1 and CerS5-silenced gastrocnemius muscle of HFD-fed mice: Panel (**A**)—insulin receptor tyrosine phosphorylation (pIR Y972); Panel (**B**)—IRS1 serine phosphorylation (pIRS S1101); Panel (**C**)—serine phosphorylation of protein kinase B/Akt (pAKT S473); Panel (**D**)—serine phosphorylation of Akt/PKB 160 kDa substrate (pAS160 S588); Panel (**E**)—protein expression of phosphoinositide 3-kinase (PI3K); Panel (**F**)—protein expression of glucotransporter 4 (GLUT4). Values are mean ± SD (*n* = 8 per group); a—*p* < 0.05 vs. LFD; b—*p* < 0.05 vs. HFD; significance by ANOVA; LFD-low-fat diet, HFD—high-fat diet. The observed molecular weight of indicated proteins is different to the theoretical one, as stated by the antibody manufacturer.

The phosphorylation state of IR at tyrosine Y972, was significantly reduced in HFD_(+/+)_ gastrocnemius compared to LFD-fed animals (*p* < 0.05). In the HFD_(-CerS1)_ muscle, the IR phosphorylation increased by 73% compared to respective values in HFD_(+/+)_ muscle (*p* < 0.05), but did not reach LFD control values. In the HFD_(-CerS5)_ gastrocnemius, the IR phosphorylation also increased significantly compared to HFD_(+/+)_ (by 24%, *p* < 0.05); although, it was lower than in HFD_(-CerS1)_ muscle (Figure 5A).

#### 3.7.2. Insulin Receptor Substrate 1 (IRS-1)

The phosphorylation of IRS-1 at S1101, increased significantly (*p* < 0.05) in HFD_(+/+)_ gastrocnemius. In both the CerS1 and CerS5 silenced muscle, a similar, significant decrease in the IRS-1 serine S1101 phosphorylation was observed, as compared to both the HFD_(+/+)_ and LFD gastrocnemius (Figure 5B).

#### 3.7.3. Phosphoinositide 3-Kinase (PI3K)

The PI3K protein content decreased by 45% in HFD_(+/+)_ (*p* < 0.05) muscle as compared to LFD control. In the gastrocnemius of HFD_(-CerS1)_ and HFD_(-CerS5)_ animals, a similar, significant increase in the level of PI3K was observed, as compared to HFD_(+/+)_ muscles, yet it did not reach LFD control values (Figure 5C).

#### 3.7.4. Protein Kinase B/Akt

The phosphorylation of Akt at serine S473, decreased significantly (by 45%) in HFD_(+/+)_ gastrocnemius as compared to LFD muscle (*p* < 0.05). Gene silencing of CerSs in muscle of HFD-fed animals (HFD_(-CerS1)_ and HFD_(-CerS5)_, respectively) led to significant increase in phosphorylation state of Akt as compared to the HFD_(+/+)_ values. It is worth noting that in the HFD_(-CerS1)_ group, the degree of Akt phosphorylation was higher than in the group HFD_(-CerS5)_ (Figure 5D).

#### 3.7.5. Protein AS160

High-fat diet consumption led to a significant decrease in AS160 phosphorylation at serine S588 in gastrocnemius muscle of HFD_(+/+)_ animals, as compared to the LFD values. In both, HFD_(-CerS1)_ and HFD_(-CerS5)_ muscle, phosphorylation state of AS160 increased significantly as compared to HFD_(+/+)_ gastrocnemius, yet did not reach LFD control values (*p* < 0.05, Figure 5E).

#### 3.7.6. Glucose Transporter Type 4 (GLUT-4)

The level of GLUT4 in HFD_(+/+)_ muscle significantly decreased as compared to LFD control (*p* < 0.05). In both groups with CerS gene silencing (HFD_(-CerS1)_ and HFD_(-CerS5)_), the level of GLUT4 increased, yet it was significantly higher than HFD_(+/+)_ values only in the case of CerS1 silencing (*p* < 0.05) (Figure 5F).

### 3.8. Insulin-Stimulated Glucose Uptake

**Figure 6:**
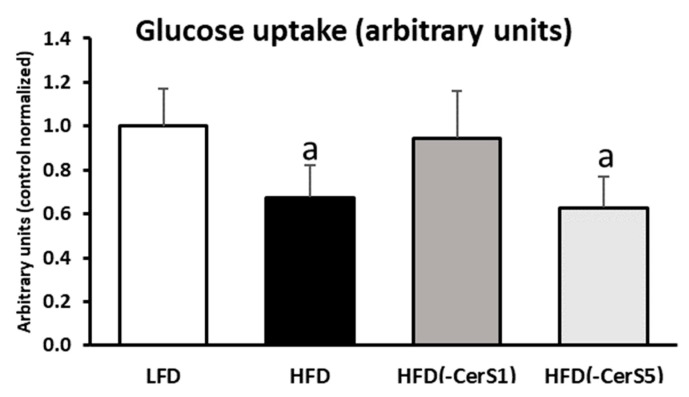
The impact of CerS1 and CerS5 gene silencing on mouse gastrocnemius muscle insulin-stimulated glucose uptake. Values are mean ± SD (*n* = 8 per group); a—*p* < 0.05 vs. LFD; significance by ANOVA; LFD-low-fat diet, HFD—high-fat diet.

High-fat feeding significantly decreased muscle insulin-stimulated glucose uptake as compared to LFD values (*p* < 0.05). Only the silencing of CerS1 in HFD_(-CerS1)_ gastrocnemius normalized muscular glucose uptake to the values indistinguishable from LFD control (*p* < 0.05). In the HFD_(-CerS5)_ group, muscular glucose uptake was similar to that observed in the HFD_(+/+)_ animals (Figure 6).

## 4. Discussion

Skeletal muscle is an important tissue crucial for both, glucose and lipid metabolism. Many studies have shown an association between the accumulation of bioactive lipids (LCACoA, DAG, and Cer) and impaired insulin signaling in skeletal muscle [13,14,15,16,17,31,32,33,34,35]. However, the role of individual lipid groups in inhibition of the insulin pathway has not been fully understood. Most of the research in this field was performed to elucidate the role of ceramide accumulation in the development of muscle insulin signaling disorders, and it has been repeatedly shown that the accumulation of ceramides in skeletal muscle is associated with the formation of insulin resistance in this tissue [36,37,38,39,40]. Recent data indicate that not all ceramides contribute equally to these disorders. Our previous work, as well as studies by other researchers, have shown that the accumulation of C18:0-Cer may be responsible for the induction of muscle insulin resistance [13,19,20,41]. However, other research groups have shown that the accumulation of C16:0-Cer may play a major role in generation of muscle insulin signaling dysfunction [42]. As the available data are inconclusive, we designed the experiment to clarify which of these ceramide species play the predominant role in the generation of disturbances in the insulin signaling pathway. To differentiate between the impact of two crucial ceramide species on skeletal muscle insulin sensitivity, we performed local electroporation-mediated silencing of genes encoding two of the most abundant isoforms of ceramide synthase in skeletal muscle: CerS1 (catalyzing C18-carbon acyl chain length ceramide synthesis) and CerS5 (catalyzing C16-carbon acyl chain length ceramide synthesis) in gastrocnemius of HFD-fed mice.

As expected, we have noticed that HFD induced whole body insulin resistance, as evidenced by increased levels of both, fasting glucose and insulin, impaired glucose and insulin tolerance, as well as increased HOMA-IR index as compared to animals fed the standard low-fat diet. Those alteration in the systemic glucose metabolism were accompanied by the inhibition of insulin signaling cascade and diminishment of glucose uptake in the gastrocnemius muscle under insulin stimulation.

To exclude the possibility that other factors than Cer could be responsible for the observed changes, we performed a comprehensive analysis of the biologically active lipids that are also relevant to the induction of insulin resistance in skeletal muscle. We noticed that HFD significantly increased the content of all the measured lipid classes, namely, LCACoA, DAG, and TAG, in the gastrocnemius muscle, but neither CerS1 nor CerS5 silencing resulted in statistically significant decrease in their content, except for ceramides. Similar data were obtained by Turpin-Nolan et al. in their work in which the muscle-specific CerS1 deficiency did not affect the content of either DAG or TAG in the muscles [19]. The silencing of the CerS1 and CerS5 genes also significantly affected insulin-stimulated signaling within insulin signal transduction pathway. In the muscles of the HFD-fed animals, the degree of IR phosphorylation of the tyrosine Y972 residue decreased significantly under insulin stimulation as compared to the LFD values, which points to the inhibition of signaling at the level of IR. Moreover, other members of the insulin signaling cascade downstream of IR also displayed adverse effect of HFD feeding, such as an increase in the inhibitory phosphorylation of serine S1101 on the insulin receptor substrate (IRS1), and reduced phosphorylation of serine S473 residue in protein kinase B (Akt/PKB). The latter is a key protein in the insulin pathway, whose insulin-stimulated phosphorylation is inhibited by the accumulation of ceramides [42,43,44,45]. It has been shown multiple times that the influence of ceramide on Akt activity is mediated by phosphatase PP2A, which reduces the degree of phosphorylation of protein kinase B (Akt) [16,46]. This leads to an inhibition of the insulin signaling cascade at the level of subsequent Akt/PKB substrate, the AS160 protein. Phosphorylation of AS160 by Akt/PKB triggers the translocation and fusion of GLUT4-containing vesicles with sarcolemma, finalizing molecular events leading to the increase in the uptake of glucose by skeletal muscle under insulin stimulation [15,16,47]. In the HFD_(+/+)_ group, we observed both a decrease in AS160 protein phosphorylation at the serine S588 residue and a significant drop of muscle glucose uptake under insulin stimulation. Disrupted muscle insulin signaling ultimately leads to decreased GLUT4 translocation to the plasma membrane and, consequently, to a decrease in muscle glucose uptake. The observed decrease in the activity of the insulin pathway in the muscles of HFD-fed animals could be attributed also to the accumulation of not only the ceramides, but also of other bioactive lipids, such as LCACoA and DAG. However, despite accumulation of LCACoA, DAG, and TAG in muscle of all HFD-fed animals, the silencing of the CerS1 or CerS5 gene significantly improved the function of the insulin signaling pathway. Nevertheless, only in the case of CerS1 silencing did we observe a significant increase in glucose uptake by skeletal muscle under insulin stimulation. This was accompanied by a decrease in the C18:0-Cer content in HFD_(-CerS1)_ muscle, and the improvement in the activity of the insulin pathway, not only at the level of the Akt/PKB protein (which is seen as the direct site of the insulin pathway regulated by ceramide), but also at the level of other proteins, including: IR, IRS, PI3K, and AS160. Those changes match the increased levels of GLUT in HFD_(-CerS1)_ muscle as compared to the HFD_(+/+)_ animals. As already mentioned, silencing of the CerS5 gene also increased the activity of the insulin pathway, yet the phosphorylation state of several proteins was muted and did not reach the levels observed for CerS1 silencing. More effective activation of the insulin pathway in the muscle of HFD_(-CerS1)_ animals compared to the HFD_(-CerS5)_ group was most noticeable for the IR and Akt proteins.

One of the first synthetized CerS inhibitor was fumonisin B. In vitro studies with the use of this compound showed that it reduces ceramide accumulation and protects muscle cells against the palmitate-induced insulin resistance [47,48]. However, this compound has only been used in in vitro studies. In vivo studies, with the use of selective CerS1 inhibitor (P053), showed that CerS1 plays an important role in regulating the oxidative capacity of mitochondria, pointing out that C18:0-Cer inhibits the β-oxidation process [20]. Furthermore, the authors of this study, contrary to our work, indicated that the inhibition of C18:0-Cer synthesis, impeded fat deposition in HFD-fed animals, but did not improve glucose metabolism and insulin sensitivity [20]. However, the use of pharmacological inhibitors is not entirely conclusive, as they induce changes at the systemic level, so there is always uncertainty about the source of the observed results.

There are also studies using animals with a deletion of ceramide synthases, not only in skeletal muscle, where the aspect of insulin sensitivity has been studied. It has been found that whole body CerS6 deletion reduced C16:0-Cer content in the adipose tissue and liver of HFD-fed animals, but did not alter the C16:0-Cer content in muscle. Nevertheless, these animals were protected against diet-induced insulin resistance [49,50]. It is worth noting, however, that the expression of CerS6 in muscle is very low compared to other isoforms of this enzymes: CerS1 and CerS5. In addition, HFD-fed animals were found to exhibit elevated liver expression of CerS6, but not of CerS5 [51], and the liver-specific deletion of CerS6 improved insulin sensitivity in HFD animals [49,50].

In our work, we observed that the silencing of the CerS1 or CerS5 gene was associated with an increase in the degree of Akt serine S473 phosphorylation, but in the HFD_(-CerS1)_ muscle this increase was higher than the HFD_(-CerS5)_ muscle. This indicates that C18:0-Cer—the main product of CerS1 gene—is likely the main ceramide responsible for the inhibition of the insulin pathway at the level of Akt/PkB. Similar results were obtained by Turpin-Nolan et al. [19], who observed an increase in the degree of phosphorylation of S473 and Thr308 residues of PKB in skeletal muscle-specific CerS1 knockout mice. The consequence of the improved functioning of the insulin pathway in the muscles of HFD_(-CerS1)_ animals is an increase in glucose uptake by these muscle as compared to HFD_(+/+)_ muscle. In HFD_(-CerS5)_ muscle, we observed an approximately 40% decrease in C16:0-Cer content as compared to the HFD_(+/+)_ muscles. Although we noticed an improvement in the function of the muscle insulin pathway in HFD_(-CerS5)_ animals, we did not observe an improvement in glucose uptake, which could indicate inhibition of the signal transduction at the level of downstream of Akt/PKB. Moreover, it is unknown how the inhibition of CerS-mediated ceramide synthesis affects other molecular pathways regulated by insulin signaling, especially protein metabolism and mitochondrial function. Further in-depth proteome-wide studies and mitochondrial functional measurements are essential to clarify this issue.

## 5. Conclusions

Significant body of evidence points to the ceramide accumulation as the major culprit in the induction of skeletal muscle insulin resistance related to caloric overload. Although the increase in total ceramide content in insulin resistant muscle was reported numerous times by different research groups, the exact molecular feature of Cer invoking adverse effects on insulin action is still under debate. The involvement of different ceramide synthase isoforms in the synthesis of Cer with specific chain length allowed us to manipulate two distinct pools of ceramide species (18-carbon chain length Cer and 16-carbon chain length Cer) through electroporation-mediated long-term shRNA silencing of individual CerS synthases in the gastrocnemius muscle of mice with HFD-induced insulin resistance. We identified a ceramide molecule that is essential for insulin inhibition. Our results indicate that C18:0-Cer is a key ceramide species involved in the induction of skeletal muscle insulin resistance, and the CerS1 could be potential important target for insulin resistance pharmacotherapy.

## Figures and Tables

**Table 1:** Glycemic parameters and free fatty acids concentration (FFA) in the plasma of LFD and HFD animals. Values are mean ± SD (*n* = 6 per group); ^a^—*p* < 0.05 vs. LFD; significance by *t*-test.

	LFD	HFD
Glucose (mg/dL)	101 ± 7.9	186 ± 25.2 ^a^
Insulin (ng/mL)	0.82 ± 0.11	1.68 ± 0.07 ^a^
OGTT AUC (×10^4^)	2.29 ± 0.14	4.02 ± 0.34 ^a^
ITT AUC (×10^4^)	0.78 ± 0.02	1.84 ± 0.16 ^a^
HOMA-IR	1.09 ± 0.09	3.17 ± 0.44 ^a^
FFA (nmol/mL)	266.8 ± 13.0	503.7 ± 18.4 ^a^

**Table 2:** Impact of CerS1 and CerS5 gene silencing on the content of individual long-chain acyl-CoA’s in mouse gastrocnemius muscle of HFD-fed animals. Values are mean pmol/mg of tissue ± SD; *n* = 8 per group; ^a^—*p* < 0.05 vs. LFD.

	C14:0-CoA	C16:0-CoA	C16:1-CoA	C18:0-CoA	C18:1-CoA	C18:2-CoA	C20:0-CoA	C22:0-CoA	C24:0-CoA	C24:1-CoA
LFD	0.11 ± 0.03	0.85 ± 0.10	0.78 ± 0.11	0.73 ± 0.08	2.59 ± 0.30	1.71 ± 0.29	0.017 ± 0.003	0.022 ± 0.003	0.028 ± 0.004	0.016 ± 0.005
HFD	0.20 ± 0.03 ^a^	0.99 ± 0.13	0.68 ± 0.07	1.20 ± 0.15 ^a^	2.69 ± 0.43	1.96 ± 0.27	0.019 ± 0.004	0.033 ± 0.005 ^a^	0.031 ± 0.005	0.009 ± 0.001 ^a^
HFD_(-CerS1)_	0.21 ± 0.02 ^a^	1.03 ± 0.23	0.71 ± 0.08	1.18 ± 0.16 ^a^	2.61 ± 0.32	2.07 ± 0.27	0.019 ± 0.002	0.035 ± 0.004 ^a^	0.032 ± 0.003	0.009 ± 0.002 ^a^
HFD_(-CerS5)_	0.21 ± 0.06 ^a^	0.99 ± 0.39	0.70 ± 0.08	1.18 ± 0.17 ^a^	2.67 ± 0.32	2.20 ± 0.32 ^a^	0.020 ± 0.002	0.035 ± 0.003 ^a^	0.031 ± 0.003	0.010 ± 0.002 ^a^

**Table 3:** Impact of CerS1 and CerS5 gene silencing on the content of individual diacylglycerol in mouse gastrocnemius muscle of HFD-fed animals. Values are mean pmol/mg of tissue ± SD; *n* = 8 per group; ^a^—*p* < 0.05 vs. LFD.

	16:0/16:0	16:0/18:0	16:0/18:1	16:0/18:2	18:0/18:0	18:0/18:1	18:0/18:2	18:1/18:1	18:2/18:2	18:0/20:0
LFD	13.41 ± 1.74	83.47 ± 10.99	32.18 ± 4.60	38.09 ± 5.47	2.10 ± 0.22	21.88 ± 2.88	0.96 ± 0.17	20.98 ± 2.14	24.91 ± 2.43	3.85 ± 0.53
HFD	16.15 ± 2.47	144.52 ± 16.79 ^a^	39.36 ± 4.67	66.52 ± 11.60 ^a^	2.92 ± 0.23 ^a^	22.90 ± 3.65	1.98 ± 0.33 ^a^	23.37 ± 2.21	34.00 ± 5.15 ^a^	7.23 ± 0.96 ^a^
HFD_(-CerS1)_	18.42 ± 2.24 ^a^	152.99 ± 8.93 ^a^	36.68 ± 5.84	68.10 ± 10.15 ^a^	2.74 ± 0.28 ^a^	24.04 ± 1.96	2.19 ± 0.29 ^a^	22.12 ± 4.19	32.27 ± 3.34 ^a^	6.88 ± 0.88 ^a^
HFD_(-CerS5)_	18.79 ± 3.12 ^a^	156.04 ± 12.08 ^a^	36.98 ± 6.09	68.87 ± 6.31 ^a^	2.81 ± 0.26 ^a^	23.98 ± 3.72	2.19 ± 0.28 ^a^	22.01 ± 1.81	31.35 ± 4.94 ^a^	7.12 ± 0.62 ^a^

**Table 4:** Impact of CerS1 and CerS5 gene silencing on the content of individual sphingolipids in mouse gastrocnemius muscle of HFD-fed animals. Values are mean pmol/mg of tissue ±SD; *n* = 8 per group; ^a^—*p* < 0.05 vs. LFD; ^b^—*p* < 0.05 vs. HFD.

	C14:0-Cer	C16:0-Cer	C18:0-Cer	C18:1-Cer	C20:0-Cer	C22:0-Cer	C24:0-Cer	C24:1-Cer	Sph	SPA	S1P
LFD	0.029 ± 0.002	1.94 ± 0.22	16.9 ± 1.4	0.56 ± 0.08	0.27 ± 0.04	0.77 ± 0.12	1.23 ± 0.14	3.8 ± 0.4	0.38 ± 0.05	0.2 ± 0.02	0.04 ± 0.01
HFD	0.034 ± 0.008	3.20 ± 0.35 ^a^	30.3 ± 3.4 ^a^	0.67 ± 0.05 ^a^	0.57 ± 0.09 ^a^	1.45 ± 0.21 ^a^	2.55 ± 0.32 ^a^	5.1 ± 0.6 ^a^	0.54 ± 0.12 ^a^	0.3 ± 0.05 ^a^	0.27 ± 0.01 ^a^
HFD_(-CerS1)_	0.033 ± 0.005	2.80 ± 0.36 ^ab^	16.5 ± 0.9 ^b^	0.40 ± 0.05 ^ab^	0.56 ± 0.09 ^a^	1.49 ± 0.19 ^a^	2.05 ± 0.24 ^ab^	4.2 ± 0.4 ^b^	0.55 ± 0.11 ^a^	0.3 ± 0.04 ^a^	0.05 ± 0.01 ^b^
HFD_(-CerS5)_	0.038 ± 0.010	1.99 ± 0.18 ^b^	26.5 ± 2.6 ^ab^	0.48 ± 0.07 ^b^	0.51 ± 0.07 ^a^	1.40 ± 0.15 ^a^	2.45 ± 0.27 ^a^	4.1 ± 0.5 ^b^	0.84 ± 0.14 ^ab^	0.3 ± 0.03 ^a^	0.31 ± 0.01 ^a^

## Data Availability

Data are contained within the article or supplementary materials.

## Supplementary Materials

The following are available online at https://www.mdpi.com/article/10.3390/cells11020206/s1, Table S1: Individual plasma free fatty acids concentration in mice fed control and high-fat diet. Values are mean ± SD (*n* = 6 per group); a—*p* < 0.05 vs. LFD; significance by unpaired *t*-test, Table S2: Impact of CerS1 and CerS5 gene silencing on the content of individual long-chain acyl-carnitines in mouse gastrocnemius muscle. Values are mean pmol/mg of tissue ±SD; *n* = 8 per group; a—*p* < 0.05 vs LFD; b—*p* < 0.05 vs HFD, Figure S1: Impact of high-fat diet (HFD) consumption on baseline normalized plasma glucose profile during oral glucose tolerance test (OGTT), Figure S2: The impact of CerS1 and CerS5 silencing on the protein expression of fatty acid trans-porters in gastrocnemius muscle of high-fat diet mice.
