# Supplementary material for: CerS1 but Not CerS5 Gene Silencing, Improves Insulin Sensitivity and Glucose Uptake in Skeletal Muscle
Source: Cells. 2022 Jan 8;11(2):206. doi: 10.3390/cells11020206 (PMC8773817; doi:10.3390/cells11020206)
Supplement: Supplementary file 1 [file cells-11-00206-s001.zip › cells-1505971-supplementary.pdf]

**Table S1.** Individual plasma free fatty acids concentration in mice fed control and high-fat diet. Values are mean ± SD (n = 6 per group); a-p < 0.05 vs. LFD; significance by unpaired t-test.

| | C14:0 | C16:0 | C16:1 | C18:0 | C18:1 | C18:2 | C20:0 | C20:2 | C20:4 | C22:0 | C22:6 | C24:0 | C24:1 |
|---|---|---|---|---|---|---|---|---|---|---|---|---|---|
| **LFD** | 7.12±1.45 | 71.60±9.36 | 6.26±1.70 | 29.98±2.67 | 70.29±7.91 | 59.06±9.76 | 1.14±0.18 | 2.28±0.37 | 9.36±1.63 | 3.83±0.30 | 1.65±0.23 | 4.11±1.04 | 0.16±0.02 |
| **HFD** | 16.74±1.23[a] | 92.80±10.81[a] | 5.38±0.48 | 61.09±4.42[a] | 141.12±6.96[a] | 138.35±6.90[a] | 4.07±0.39[a] | 2.61±0.16 | 19.44±1.14[a] | 11.28±0.79[a] | 2.22±0.19[a] | 8.21±0.58[a] | 0.38±0.07[a] |

**Table S2.** Impact of CerS1 and CerS5 gene silencing on the content of individual **long-chain acyl-carnitines** in mouse gastrocnemius muscle. Values are mean pmol/mg of tissue ±SD; n=8 per group; a –p<0.05 vs LFD; b –p<0.05 vs HFD.

| | C14:0-carnitine | C16:0-carnitine | C18:1-carnitine | C18:0-carnitine |
|---|---|---|---|---|
| **LFD** | 1.47±0.18 | 2.46±0.22 | 6.94±0.54 | 0.72±0.09 |
| **HFD** | 1.19±0.18[a] | 2.93±0.33 | 7.31±0.51 | 1.23±0.14[a] |
| **HFD(-CerS1)** | 1.45±0.06[b] | 3.08±0.35[a] | 7.93±1.01 | 1.44±0.21[ab] |
| **HFD(-CerS5)** | 1.48±0.06[b] | 3.46±0.49[ab] | 7.97±1.03 | 1.31±0.09[a] |

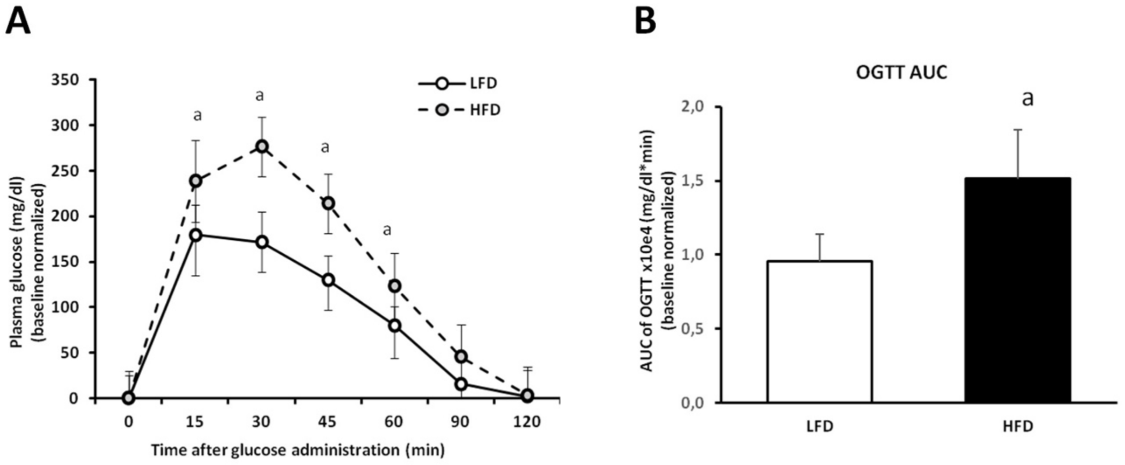


**Figure S1**. Impact of high-fat diet (HFD) consumption on baseline normalized plasma glucose profile during oral glucose tolerance test (OGTT). Panel A— baseline normalized plasma glucose profile during OGTT; Panel B—area under the baseline-normalized plasma glucose curve (AUC) for OGTT; Values are mean ± SD (n = 6 per group); *-p < 0.05 vs. LFD; significance by unpaired t-test.

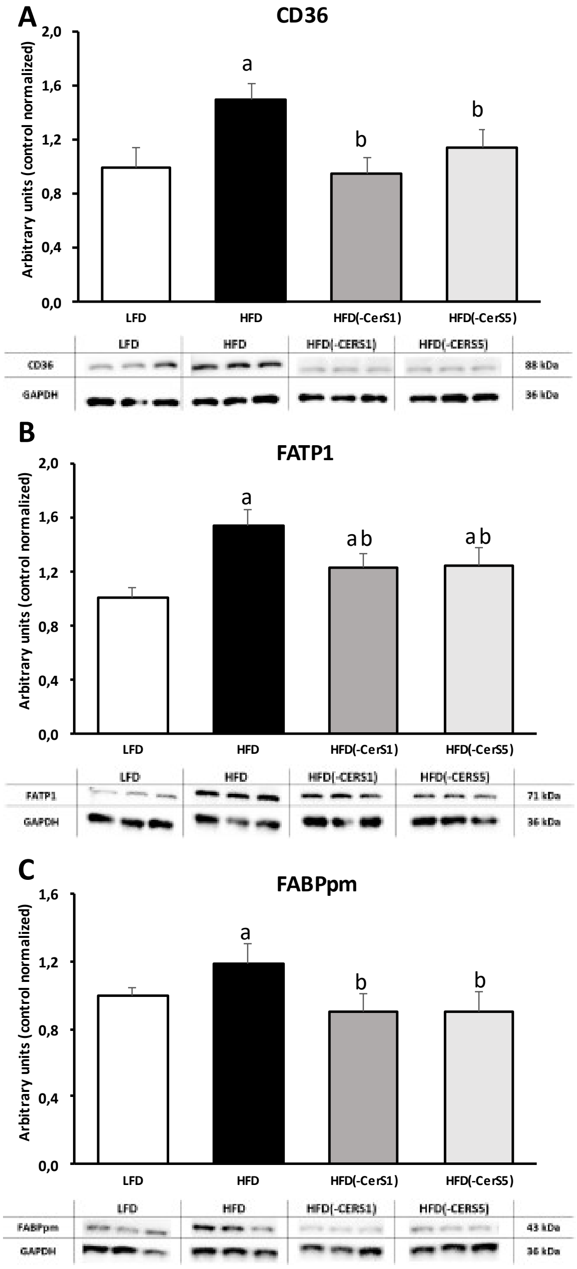


**Figure S2**. The impact of CerS1 and CerS5 silencing on the protein expression of fatty acid transporters in gastrocnemius muscle of high-fat diet mice. Panel A-protein expression of CD36; Panel B-protein expression of FATP1; Panel C-protein expression of FABPpm. Values are mean ±SD n=8 per group; a –$p<0.05$ vs LFD; b –$p<0.05$ vs HFD; significance by ANOVA; LFD-low-fat diet, HFD—high-fat diet. * The observed molecular weight of indicated proteins is different to the theoretical one, as stated by the antibody manufacturer.
